# Bilateral granulomatous panuveitis in two patients with T-cell type of chronic active Epstein-Barr virus infection

**DOI:** 10.1186/s12886-019-1090-5

**Published:** 2019-03-29

**Authors:** Hiroyuki Takahashi, Hiroshi Takase, Ayako Arai, Manabu Mochizuki, Kyoko Ohno-Matsui

**Affiliations:** 1 Department of Ophthalmology & Visual Science Graduate School of Medical and Dental Sciences, Tokyo Medical and Dental University (TMDU), 1-5-45, Yushima, Bunkyo-Ku, Tokyo, 113-8519 Japan; 2 Department of Molecular Genetics of Hematology, Graduate School of Medical and Dental Sciences, Tokyo Medical and Dental University (TMDU), Tokyo, Japan

**Keywords:** Chronic active Epstein-Barr virus infection, Uveitis, PCR, Aqueous humor

## Abstract

**Background:**

To report 2 cases of bilateral granulomatous panuveitis accompanied by chronic active Epstein-Barr virus infection (CAEBV).

**Case presentation:**

Case 1 was a 38-year-old man who had a history of bilateral mild panuveitis who was diagnosed with CAEBV. Fifteen months later, a severe bilateral granulomatous panuveitis developed. White infiltrates covered the optic disc and all the retinal vessels of the right eye, and white nodules were seen along the retinal veins and arteries of the left eye. Case 2 was a 34-year-old man with bilateral panuveitis showing mutton-fat keratic precipitates and diffuse vitreous opacity in both eyes. A snow ball-like vitreous opacity was present in the right eye. Systemic investigations revealed the presence of CAEBV. In both cases, a comprehensive polymerase chain reaction (PCR) analyses of the aqueous humor detected significant copy numbers of EBV-DNA. The intraocular inflammation did not respond to steroid, methotrexate, and other immunosuppressive therapies, but was ameliorated after hematopoietic stem cell transplantation with preceding chemotherapy and low-dose total body irradiation in both cases.

**Conclusion:**

Granulomatous panuveitis can develop in eyes with CAEBV as a primary symptom. Ophthalmologists should rule out CAEBV when EBV-DNA is positive in the intraocular fluids of steroid-resistant panuveitis.

## Background

There are many reports implicating Epstein-Barr virus (EBV) as the cause of intraocular inflammation. EBV-DNA can be detected in the intraocular fluids with various uveitis entities [1–3], and it is possible that EBV can cause necrotizing retinitis in immunocompromised patients [4, 5]. However, there is no direct evidence that EBV can cause uveitis in immunocompetent patients, i.e., does EBV directly infect the ocular tissue or EBV-infected cells infiltrate the tissue.

Chronic active EBV infection (CAEBV) is a rare systemic disorder characterized by persistent or recurrent systemic inflammation such as fever, liver dysfunction, and vasculitis with clonal proliferation of EBV-infected T- or NK-cells in the peripheral blood. Most cases have been reported from east Asia especially Japan [6]. However, reports are increasing from all over the world since CAEBV was classified as being associated with mature T- or NK-cell neoplasms in the WHO classification of tumors of hematopoietic and lymphoid tissues revised in 2017 [7]. CAEBV is chemotherapy-resistant, and hematopoietic stem cell transplantation (HSCT) has been the only promising treatment for a cure. Otherwise, CAEBV progresses to highly malignant lymphoma, multiple organ failure, or hemophagocytic lymphohistiocytosis (HLH) during course and can be fatal. Thus, an early and accurate diagnosis is indispensable.

The ocular involvement of CAEBV has not been well recognized [8–10], and only one article confirmed an EBV infection of T- or NK- cells by genomic analysis [8]. We report 2 cases of uveitis accompanied by CAEBV. Both cases exhibited steroid-resistant granulomatous panuveitis bilaterally as the primary symptom of CAEBV, and the analysis of the aqueous humor (AqH) by polymerase chain reaction (PCR) revealed the presence of EBV-DNA. Although the ocular signs and symptoms did not respond to systemic chemotherapies, HSCT ameliorated the ocular complications.

### Case presentations

#### Case 1

A 38-year-old man, who had been treated for mild bilateral panuveitis with topical steroid eye drops for 2 years, experienced severe fatigue and visited an internal medicine physician. Due to severe heart failure and infiltration of T-cells infected with EBV to the heart muscle, he was referred to the Department of Hematology of Tokyo Medical and Dental University (TMDU) Hospital. The peripheral blood mononuclear cells (PBMCs) obtained from patients were isolated by density gradient centrifugation using Separate-L (Muto Pure Chemical, Tokyo, Japan) and were sorted into CD19-, CD4-, CD8-, or CD56-positive fractions using antibody-conjugated magnetic beads (Miltenyi Biotec, Bergisch Gladbach, Germany; 130–050-301, 130–045-101, 130–045-201, 130–090-875). After that, the EBV DNA levels in the whole blood and each fraction were evaluated by real-time PCR using the TaqMan System (Applied Biosystems, Foster City, CA) [11]. The EBV-DNA load was determined to be 1.2 × 10^5^ copies/μg DNA in the whole blood, and 1.7 × 10^4^ copies/μg DNA in the CD4-positive T-cell fraction. Southern blot analysis for EBV-terminal repeat revealed the clonality of the EBV-infected cells [12]. From the clinical, serological, and pathological findings, he was diagnosed with CAEBV.

He was then referred to our clinic to screen for ocular involvements. Our examination showed that his best-corrected visual acuity (BCVA) was 20/16, and 1+ cells were detected in the anterior vitreous in both eyes. Systemic work-up for the differential diagnosis of uveitis including blood tests, tuberculin skin tests, and the chest X-rays did not suggest any specific type of uveitis such as sarcoidosis, ocular tuberculosis, syphilis, or human T-cell leukemia virus type 1 (HTLV-1) uveitis. He was being treated with 0.1% betamethasone eye drops 4 times a day which was continued.

**Fig. 1:**
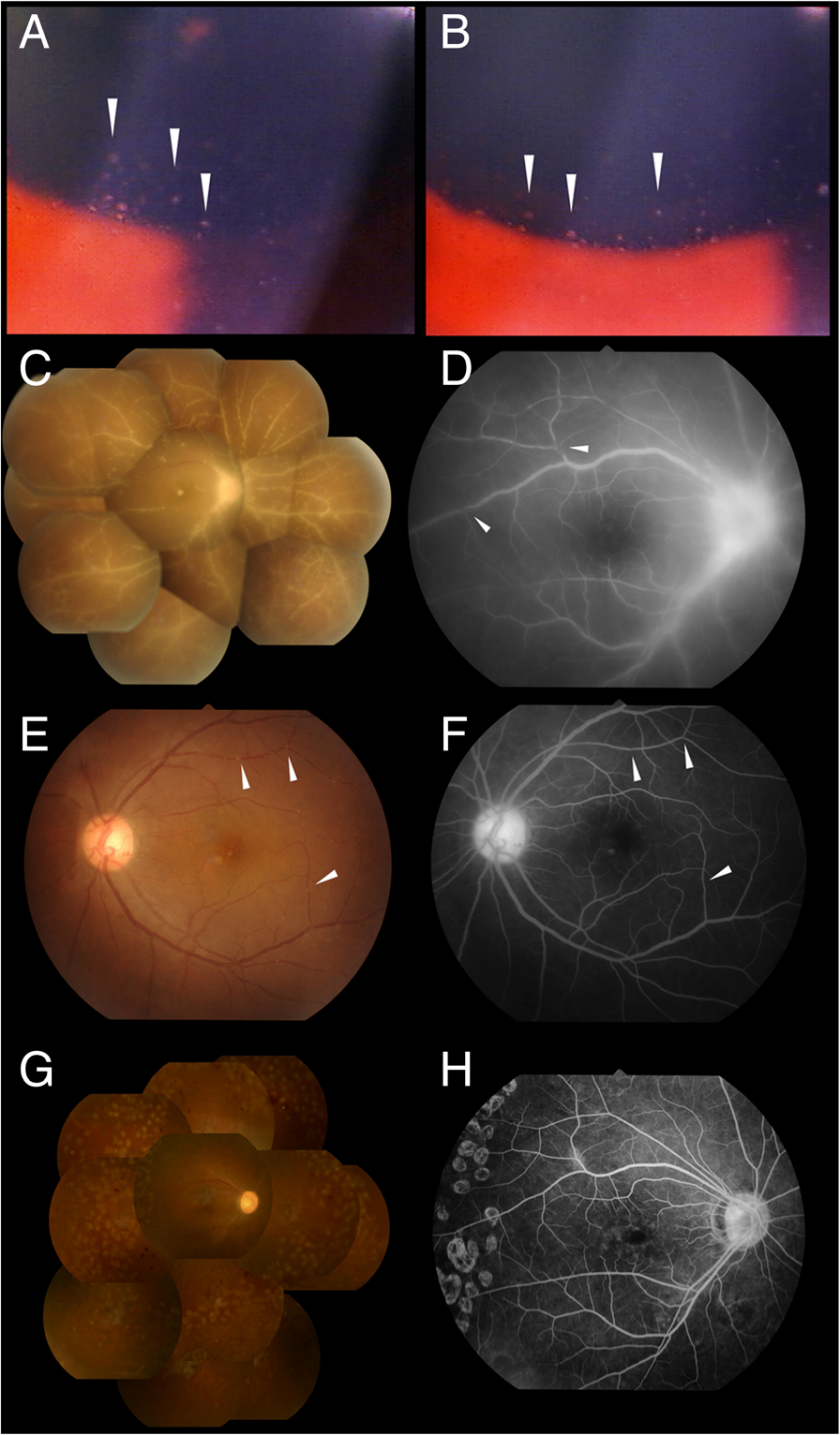
A 38-year-old man with chronic active Epstein-Barr virus infection. Mutton fat keratic precipitates (**a**, **b**. arrowhead) and cellular infiltration are present in the anterior chamber of both eyes (**a**; right eye, **b**; left eye). The optic disc, fovea, and all of the retinal vessels are covered with white infiltrates (**c**), and fluorescein angiography (FA) showed hyperfluorescence of the optic disc, dye leakage from the retinal vessels, and stenosis of the retinal vessels (**d**). The left fundus has several white nodules along the retinal arteries (arrowhead), and FA show that the retinal vessels are narrowed by the nodular lesions (**e** and **f**). After cord blood transplantation, signs of intraocular cell infiltration completely disappeared in 8 weeks. Because an epiretinal membrane was present and capillary non-perfused areas developed, pars plana vitrectomy and endo-photocoagulation were performed. Color fundus photograph (**g**) and FA (**h**) of the right eye show scars of the laser photocoagulation

Systemic immunotherapy: prednisolone 1–2 mg/kg/day and cyclosporine A 3 mg/kg/day was started for the CAEBV. The preparation for allogeneic stem cell transplantation was started simultaneously. However, 15 months later, he experienced a sudden visual disturbance of his right eye. His BCVA was decreased to 20/32. Slit-lamp ophthalmoscopy showed mutton-fat keratic precipitates (KPs), 2+ cells in the anterior chamber, and 2+ cells in the anterior vitreous of both eyes (Fig. 1a and b). In the right eye, the optic disc, fovea, and all of the retinal vessels were covered with white infiltrates (Fig.1c). In the left eye, several white nodules were seen along the retinal veins and arteries (Fig. 1e). Fluorescein angiography (FA) demonstrated hyper-fluorescence of the optic disc, dye leakage from the retinal vessels, and stenosis of the retinal vessels of the right eye (Fig.1d). The retinal vessels of the left eye were similarly narrowed by nodular lesions (Fig. 1f).

The right anterior chamber was tapped, and DNA was extracted from AqH sample using a DNA Mini kit (Qiagen, Valencia, CA). The DNA was then processed for multiplex PCR targeting specific genomic sequences of all 8 types of human herpes viruses, e.g. herpes simplex virus type-1 and 2, varicella-zoster virus, cytomegalovirus, EBV, and other pathogens including *Toxoplasma gondii* and *Mycobacterium tuberculosis*. Real-time PCR for ribosomal DNA of bacteria and fungi was also performed. The multiplex PCR and real-time PCR were performed using a LightCycler 480 II instrument (Roche, Basel, Switzerland). Primers, probes, and PCR conditions used for human herpes viruses, *Toxoplasma gondii*, *Mycobacterium tuberculosis*, and ribosomal DNA of bacteria and fungi have been described previously [13–17]. The results showed that only EBV-DNA was detected in the AqH with a load of 3.38 × 10^4^ copies/ml. To determine the concentrations of interleukin (IL)-6 or IL-10 in the AqH, 50 μL of AqH supernatant was applied for enzyme-linked immunosorbent assay, according to the manufacturer’s instructions (Invitrogen, Camarillo, CA). As a result, IL-6 concentration was over 1000 pg/ml, whereas that of IL-10 was below the detectable level. PCR for T cell receptors (TCR) and immunoglobulin heavy chain (IgH) gene rearrangements was negative for clonality.

Because of the presence of CAEBV and detection of EBV-DNA in the AqH, we diagnosed the ocular involvements as uveitis related to CAEBV. The patient was treated with a sub-Tenon injection of 20 mg/0.5 ml of triamcinolone acetonide (STTA) and intravitreal injections of methotrexate (400 μg/100 μl) together with 40 mg of systemic prednisolone and 100 mg of cyclosporine daily. However, the ocular lesions did not respond. Finally, he underwent cord blood transplantation (CBT) using reduced-intensity conditioning in the Department of Hematology: fludarabine (Flu, 100 mg/m^2^, 4 doses), melphalan (Mel,70 mg/m^2^, 1 dose), antithymocyte globulin (1.25 mg/kg/day for 2 days), and low-dose total body irradiation (4 Gy). Although EBV-positive T-cells were not eradicated from the peripheral blood: the viral load of the whole blood was 4.3 × 10^3^ copies/μg DNA, the intraocular inflammation completely disappeared in 8 weeks. We performed pars plana vitrectomy and endo-photocoagulation for epiretinal membrane and non-perfused area of the retina (Fig. 1g and h). Since then, the intraocular inflammation has been inactive for 5 years, and his last BCVA was 20/125 OD and 20/20 OS.

#### Case 2

**Fig. 2:**
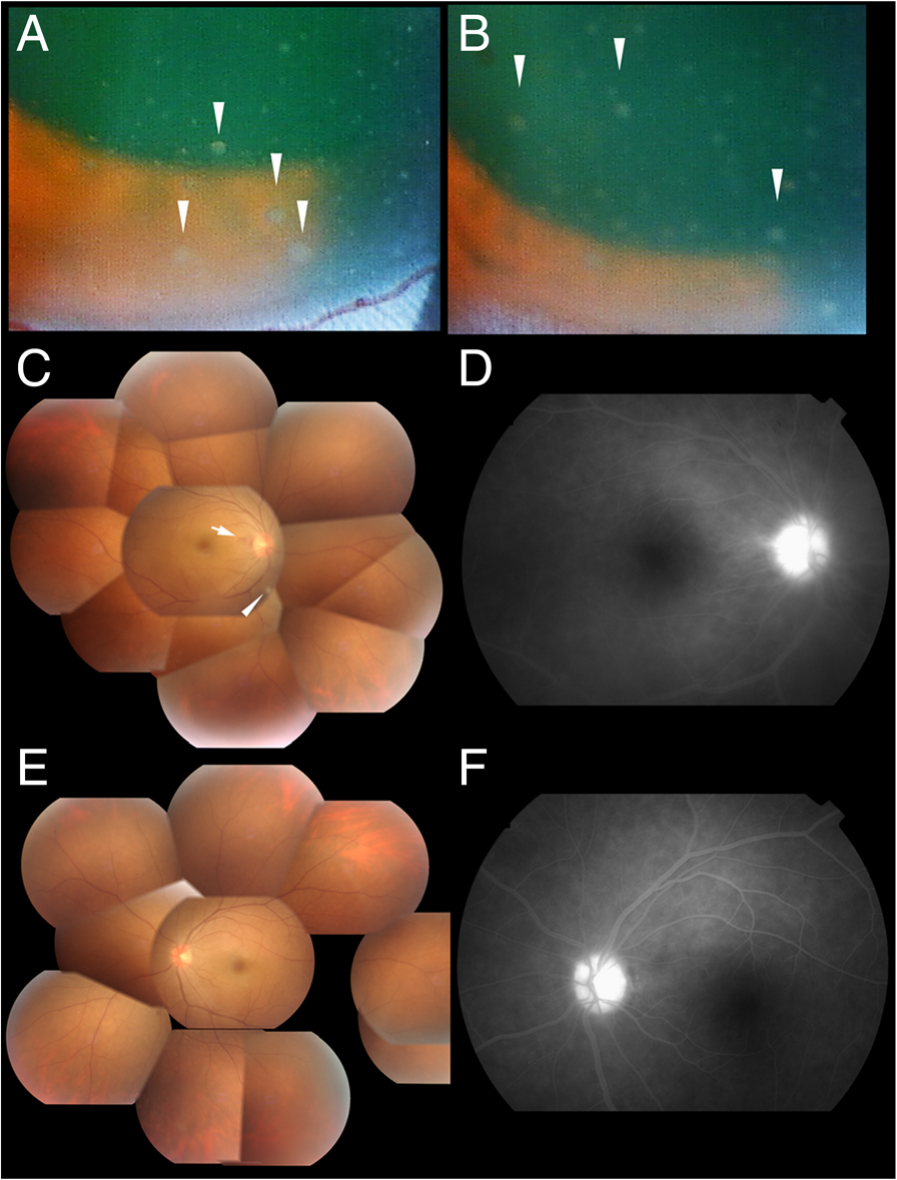
A 34-year-old man with chronic active Epstein-Barr virus infection. Mutton fat keratic precipitates (**a**, **b**. arrowhead) and cellular infiltrations are present in the anterior chamber of both eyes (**a**; right eye, **b**; left eye). His right eye has diffuse vitreous opacity, snow ball-like vitreous opacity (arrowhead), and disc hemorrhage (arrow) (**c**). Fluorescein angiography (FA) of the right eye show hyperfluorescence of the optic disc and mild dye leakage from the retinal vessels (**d**). His left eye shows diffuse vitreous opacity (**e**), and FA demonstrates hyperfluorescence of the optic disc and mild dye leakage from the retinal vessels (**f**)

A 34-year-old man who had been treated with steroid eye drops for 5 months due to panuveitis was referred to our clinic. At his initial examination, his BCVA was 20/16 in both eyes. Slit-lamp examination showed mutton fat KPs and 1+ cells in the anterior chamber and the anterior vitreous of both eyes (Fig. 2a and b). Diffuse vitreous opacities were present in both eyes, and a snow ball-like vitreous opacity and disc hemorrhage were seen in the right eye by indirect ophthalmoscopy (Fig. 2c and e). FA demonstrated hyperfluorescence of the optic disc, and fuzzy dye leakage from the retinal capillaries of both eyes (Fig. 2d and f). Systemic examinations including blood tests, chest X-rays, and thoracoabdominal computed tomography (CT) scan enhanced with iodine were performed. Although the blood tests and chest X-rays did not suggest any specific uveitis entities, CT scan revealed the presence of splenomegaly and lymphadenopathy. We then consulted the Department of Hematology, and their examination showed 1.2 × 10^5^ copies/μg of EBV-DNA in his peripheral whole blood. EBV infection of the CD4-positive T-cells and their clonal proliferation were confirmed by Southern blotting for EBV-terminal repeat. [12] Endoscopic biopsies revealed that EBV infected T-cells had infiltrated his lungs and gastric mucous membrane. Concurrently, a tap of the anterior chamber of the right eye, and multiplex PCR followed by real-time PCR was performed as described in case 1, and EBV-DNA was detected in the AqH with 3.23 × 10^4^ copies/ml. Cytokine measurements by ELISA (Invitrogen, Camarillo, CA) detected 386 pg/ml of IL-6 but IL-10 was undetectable. PCR for TCR and IgH gene rearrangement was negative for monoclonality.

As in Case 1, we diagnosed the ocular involvements as uveitis related to CAEBV, and we treated the patients with 0.1% betamethasone eye drops 4 to 6 times a day, applying one drop each time, followed by STTA, but the intraocular inflammation did not respond. Finally, allogeneic bone marrow transplantation (BMT) from one allele-mismatch unrelated donor using reduced-intensity conditioning was performed as follows; Flu (100 mg/m^2^, 4 doses), Mel (80 mg/m^2^), and low-dose total body irradiation (4 Gy). EBV-DNA load in the whole blood was 3.9 × 10^4^ copies/μg DNA, and EBV-positive T-cells were not eradicated from the peripheral blood. However, the disease became inactive. The ocular lesions completely resolved after the HSCT. The BCVA at 6 months after BMT was 20/16 OU.

## Discussion

This is the first report to describe in detail the uveitis associated with CAEBV. The specific findings, especially in Case 1, were white infiltrates covering the optic disc, fovea, and the entire retinal vessels in the right eye, and the white nodules seen along with the retinal arteries in the left eye. In addition, these two cases shared some common features, i.e., young male adults, and EBV infection of peripheral CD4-positive T-cells. The elevation of IL-6 and not IL-10, and presence of EBV-DNA in the AqH were common findings of these two patients. A collection of the findings of similar types of patients is indispensable to determining the relationship between these factors and the development of uveitis.

The presence of EBV-DNA in the AqH indicates an intraocular invasion of EBV-infected cells or direct infection of EBV on the ocular tissues. Furthermore, EBV-DNA can be detected in the plasma in some cases of CAEBV. Because the infection type of EBV is latent without replication of the virus in CAEBV [18], EBV-DNA in the plasma is considered to be the fragments and not the viral particles. Thus, the vitreous EBV-DNA could be DNA fragments from the peripheral blood. Further studies including histological examination will be necessary to clarify the origin of EBV-DNA in the AqH.

A recent nationwide survey in Japan reported that 4% (4/100) of patients had uveitis (unpublished data). On the other hand, an earlier retrospective observational study of 108 CAEBV patients had no case of uveitis. Uveitis may have been overlooked in CAEBV, and this complication should be informed to clinical hematologists.

The only promising treatment for resolving the EBV-infected neoplastic cells in CAEBV has been HSCT [19]. Although recent studies suggest some degree of effectiveness of valganciclovir for replication of EBV [20–22], there have been no studies which found the effectiveness of valganciclovir or ganciclovir for CAEBV. In the present 2 cases, EBV-infected cells could not be eradicated, but the disease activities: inflammatory symptoms including uveitis were resolved after HSCT. Therefore, we recommend that conditioning chemotherapy and low-dose total body irradiation prior to the transplantation may ameliorate the local and systemic inflammations possibly by suppressing production of inflammatory molecules such as cytokines from EBV-positive T-cells. Because Case 2 still has EBV-positive T cells in the peripheral blood, we are keeping a close observation on the patient for possible recurrences of the intraocular inflammation.

## Conclusions

In conclusion, granulomatous panuveitis can develop in patients with CAEBV as a primary symptom. Clinical ophthalmologists should rule out CAEBV when EBV-DNA is positive in the intraocular fluids of steroid-resistant panvuveitis.

## Abbreviations

AqH: Aqueous humor
BCVA: Best-corrected visual acuity
BMT: Bone marrow transplantation
CAEBV: Chronic active Epstein-Barr virus infection
CBT: Cord blood transplantation
CT: Computed tomography
DNA: Deoxyribonucleic acid
EBV: Epstein-Barr virus
ELISA: Enzyme-linked immunosorbent assay
FA: Fluorescein angiography
Flu: Fludarabine
HLH: Hemophagocytic lymphohistiocytosis
HSCT: Hematopoietic stem cell transplantation
HTLV-1: Human T-cell leukemia virus type 1
IgH: Immunoglobulin heavy chain
IL: Interleukin
KP: Keratic precipitate
Mel: Melphalan
PCR: Polymerase chain reaction
PBMCs: Peripheral blood mononuclear cells
STTA: Sub-Tenon injection of triamcinolone acetonide
TCR: T cell receptor
